# Modulation of the glyoxalase system in the aging model *Podospora anserina*: effects on growth and lifespan

**DOI:** 10.18632/aging.100251

**Published:** 2010-12-30

**Authors:** Christian Q. Scheckhuber, Sandra J. Mack, Ingmar Strobel, Filomena Ricciardi, Suzana Gispert, Heinz D. Osiewacz

**Affiliations:** ^1^ Faculty for Biosciences & Cluster of Excellence Macromolecular Complexes, Molecular Developmental Biology, Goethe University, D-60438 Frankfurt am Main, Germany; ^2^ Section Molecular Neurogenetics, Dept. Neurology, Goethe University Medical School, D-60590 Frankfurt am Main, Germany

**Keywords:** Podospora anserina, aging, lifespan, glycation, glucose, methylglyoxal, advanced glycation end products

## Abstract

The eukaryotic glyoxalase system consists of two enzymatic components, glyoxalase I (lactoylglutathione lyase) and glyoxalase II (hydroxyacylglutathione hydrolase). These enzymes are dedicated to the removal of toxic α-oxoaldehydes like methylglyoxal (MG). MG is formed as a by-product of glycolysis and MG toxicity results from its damaging capability leading to modifications of proteins, lipids and nucleic acids. An efficient removal of MG appears to be essential to ensure cellular functionality and viability. Here we study the effects of the genetic modulation of genes encoding the components of the glyoxalase system in the filamentous ascomycete and aging model Podospora anserina. Overexpression of PaGlo1 leads to a lifespan reduction on glucose rich medium, probably due to depletion of reduced glutathione. Deletion of PaGlo1 leads to hypersensitivity against MG added to the growth medium. A beneficial effect on lifespan is observed when both PaGlo1 and PaGlo2 are overexpressed and the corresponding strains are grown on media containing increased glucose concentrations. Notably, the double mutant has a ‘healthy’ phenotype without physiological impairments. Moreover, PaGlo1/PaGlo2_OEx strains are not long-lived on media containing standard glucose concentrations suggesting a tight correlation between the efficiency and capacity to remove MG within the cell, the level of available glucose and lifespan. Overall, our results identify the up-regulation of both components of the glyoxalase system as an effective intervention to increase lifespan in P. anserina.

## INTRODUCTION

The filamentous ascomycete *Podospora anserina* is an established model system to unravel the complex network of pathways controlling organismal aging [1-4]. In contrast to most other filamentous fungi, *P. anserina* wild type isolates display a limited lifespan of only a few weeks. During this period of time the morphology and physiology of growing cultures changes. Senescent cultures are characterized by a decreased growth rate, loss of fertility and an increased pigmentation, a phenotype that collectively is termed the ‘senescence syndrome’ [5].

Aging research on *P. anserina* and other model systems involves the identification of cellular pathways which contribute to an increased functional period of time (‘healthspan’) [3, 6]. In *P. anserina* several pathways have been identified which indeed lead to the phenomenon of ‘healthy aging’. These include the reduction of mitochondrial fission [7, 8], modulation of a gene encoding a SAM-dependent O-methyltransferase [9-11] and increase of the mitochondrial PaLON protease [12]. In the latter study it was shown that mitochondrial protein modifications (i. e., carbonylation and carboxymethylation) increase during aging. Carboxymethylation is an example for an important modification which involves the formation of ‘advanced glycation end products’ (AGEs). The mechanism leading to the formation of these compounds appears to be clear: first, a sugar aldehyde or ketone reacts with an amino group (e.g., a proteinaceous amino acid) in a non-enzymatic reaction. The resulting intermediate is a Schiff base which can be rearranged to fructosamine (i. e., Amadori product). Further modifications of the Amadori product can involve dehydration, cyclization and condensation reactions, giving eventually rise to AGEs [13]. AGEs are described as being highly detrimental because they have the potential to cause irreversible damage to proteins, lipids and nucleic acids [14-17].

The highly reactive α-oxoaldehyde methylglyoxal (MG) is a key compound involved in the generation of AGEs. It is mainly generated as a by-product of glycolysis from the triose phosphate intermediates dihydroxyacetone phosphate and glyceraldehyde-3-phosphate [18]. To counteract the deleterious effects of MG and other toxic reactive carbonyl and α-oxoaldehyde glycating agents, organisms contain an enzymatic defence system comprised of glyoxalase I (lactoyl-glutathione lyase) and glyoxalase II (hydroxyacyl-glutathione hydrolase) [14]. α-Oxoaldehydes and glutathione form spontaneously a hemithioacetal that is converted into *S*-2-hydroxyacylglutathione derivatives by glyoxalase I. Glyoxalase II subsequently hydrolyses this compound to GSH and 2-hydroxycarboxylates like lactate.

The glyoxalase system, its capacity to detoxify compounds involved in the generation of AGEs and an effect on aging have recently been the focus of several studies. For example, in the nematode *Caenorhabditis elegans* it was shown that activity of glyoxalase I declines during aging [19]. In old animals, both AGE and oxidative markers were found to be significantly increased when compared to young animals. Modulation of glyoxalase I activity was found to affect lifespan of transgenic worms (i. e., overexpression of *CeGly1* leads to an increased mean lifespan of +29% whereas strains with a down-regulated *CeGly1* are short-lived [-52 %]). In mammalian cell cultures (i.e., WI-38 fibroblasts) it was demonstrated that the load of AGE-modified proteins increases during replicative senescence [20]. Proteins of the cytoplasm and mitochondria were found to be affected, some of the candidates involved in fundamental cellular functions like energy metabolism and quality control of proteins. Similar to *C. elegans*, increased age was associated with decreased glyoxalase I activity. These findings suggest an important role of the glyoxalase detoxification system in the age-related increase of damaged proteins.

Here we report data demonstrating a clear impact of the glyoxalase system on the aging process of *P. anserina*. Transgenic strains overexpressing the genes encoding the *P. anserina* homologs of glyoxalase I (*PaGlo1*) and /or glyoxalase II (*PaGlo2*) and a *PaGlo1* deletion mutant (Δ*PaGlo1*) were generated, verified and characterized. Our data demonstrate that the glyoxalase system is an efficient component in the network of pathways affecting aging and lifespan of *P. anserina.*Enhancing the system via genetic manipulation is effective in slowing down aging leading to lifespan extension.

## RESULTS

### Identification of *PaGlo1* and *PaGlo2* in the genomic sequence of *P. anserina*

**Figure 1.**
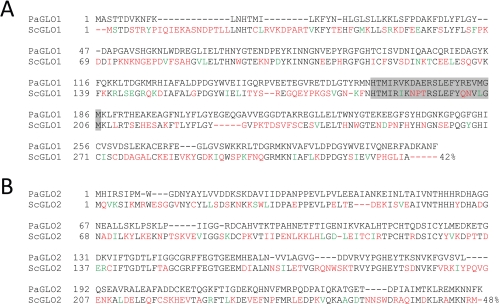
Homology analysis between *P. anserina* and yeast proteins of the glyoxalase system **A** Comparison of the amino acid sequences of glyoxalase I proteins from *P. anserina* (PaGLO1, UniProt accession number B2AQW8) and *S. cerevisiae* (ScGLO1, UniProt accession number P50107). Signature glyoxalase I sequences are indicated with gray boxes and were identified by utilizing the Prosite database. **B** Comparison of the amino acid sequences of glyoxalase I proteins from *P. anserina* (PaGLO2, UniProt accession number B2B554) and *S. cerevisiae* (ScGLO2, UniProt accession number Q05584). After the yeast sequences the amino acid identity relative to the *P. anserina* proteins is shown. Non-homologous and homologous amino acids are indicated in red and green colour, respectively.

The published amino acid sequences of *Saccharomyces cerevisiae* GLO1 (UniProt accession number P50107) and GLO2 (UniProt accession number Q05584) were used to search the genomic sequence of *P. anserina* [21] for the corresponding homologs via the BlastX (protein sequence vs. nucleotide sequence) algorithm [22]. For each protein, one homolog, designated PaGLO1 and PaGLO2, respectively, was identified. The sequence homology of PaGLO1 and PaGLO2 to their *S. cerevisiae* counterparts is strongly conserved, displaying 42% and 48% sequence identity, respectively (Figure 1). In the PaGLO1 sequence, a signature glyoxalase I motif was identified (amino acid positions 165-186) by an *in silico* analysis using Expasy Prosite. It is located in the central region of the protein and contains a conserved histidine residue which is implicated to bind the zinc cofactor in human GLO1 [23] (Figure 1).

The available genome sequence of *P. anserina*is derived from wild type strain ‘S’. Since all experiments in the current study were performed with a different wild type strain, ‘s’, the two genes from this strain were sequenced and the gene sequences were compared with the published sequences. No differences within the genes between the two strains were identified. The accession numbers assigned by UniProt are B2AQW8 (PaGLO1) and B2B554 (PaGLO2), respectively.

### Generation and verification of transgenic *PaGlo1* and *PaGlo2 P. anserina* strains

**Figure 2.**
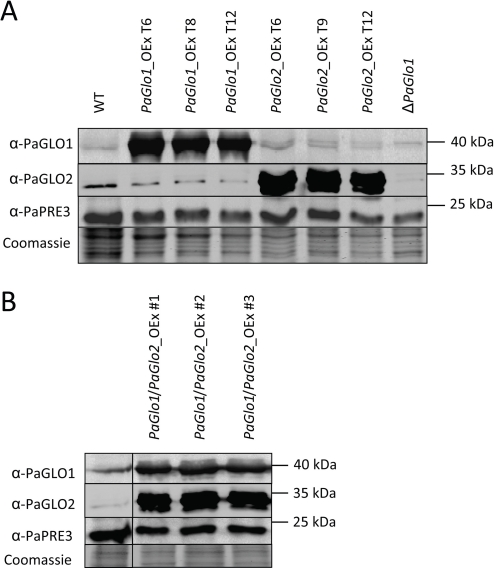
Immunodetection analysis of PaGLO1 and PaGLO2 levels in the wild type (WT) and transgenic glyoxalase strains. A Total protein extracts isolated from WT, single *PaGlo1*- and *PaGlo2-* overexpression mutants (three independent isolates from each *PaGlo1*_OEx and *PaGlo2*_OEx, respectively) and the deletion strain Δ*PaGlo1* were analyzed using antibodies against PaGLO1 and PaGLO2 after transfer to a PVDF membrane. **B**Total protein extracts isolated from WT and double *PaGlo1*/*PaGlo2* overexpression mutants (three independent isolates) were similarly analyzed. Detection of the cytosolic protein PaPRE3 (β1-subunit of the 20S proteasome) and protein staining of the transfer membrane with Coomassie were performed as loading controls. Accession numbers: PaGLO1: B2AQW8 (UniProt); PaGLO2: B2B554 (UniProt); PaPRE3: Pa_5_4560 (*Podospora anserina*sequencing consortium).

After the identification of *PaGlo1* and *PaGlo2* we set out to characterize the glyoxalase system of *P. anserina* by creating and analyzing various transgenic glyoxalase strains (i. e., single *PaGlo1* overexpression, single *PaGlo2* overexpression, double *PaGlo1*/*PaGlo2* overexpression and *PaGlo1* deletion). Plasmids for modulation of the glyoxalase system in *P. anserina* were constructed, verified and transformed into wild type protoplasts. Three independent putative overexpression transformants for *PaGlo1* and *PaGlo2,* respectively *,* were selected on hygromycin B-containing medium. Transformants in which *PaGlo1* is deleted were selected by growth on phleomycin-supplemented medium. *PaGlo1*/*PaGlo2*_OEx double mutants were generated by crossing the single overexpression mutants and selection of progeny in which recombination resulted in the combination of the *PaGlo1* and *PaGlo2* overexpression cassettes in one nucleus. The complete integration of the *PaGlo1* or *PaGlo2* expression cassettes into the genome and the deletion of *PaGlo1* were verified via Southern-blot analyses (data not shown). Subsequently, Western-blot analyses were performed using newly generated specific polyclonal peptide antibodies against PaGLO1 and PaGLO2, respectively (Figure 2). All *PaGlo1*_OEx and *PaGlo2*_OEx transformants were found to contain significantly elevated levels of PaGLO1 and PaGLO2, respectively (Figure 2 A). As expected, the PaGLO1 protein is not detectable in the Δ*PaGlo1* strain (Figure 2 A). However, in some transformants (e.g., *PaGlo2*_OEx strains) a further signal is visible, slightly above PaGLO1. This additional band is possibly due to unspecific cross-reaction of the polyclonal PaGLO1 antibody. The successful selection of *PaGlo1*/*PaGlo2*_

OEx double mutants was verified by the demonstration of strongly elevated levels of both PaGLO1 and PaGLO2 in the corresponding strains (Figure 2 B).

### Growth tests on media supplemented with additional glucose and MG

Next we tested whether altered expression of genes encoding components of the glyoxalase system has an effect on growth rate under different conditions. It is known that elevated levels of glucose lead to the formation of increased MG levels [24]. Therefore we first investigated growth of the transformants on medium supplemented with elevated levels of glucose (2% instead of 1%). No changes of the mean growth rate were found when the different genetically modified strains were grown on synthetic medium (PASM) with 2% glucose compared to the wild type (Table 1). It is important to note that these types of experiments need to be performed simultaneously by growing the strains to be compared in one set of experiment. This is crucial because even small changes in conditions (e.g., media composition) can lead to differences in reads-outs.

In a second set of experiments, we challenged the different strains with elevated levels of MG. Growth rate determinations were performed on PASM containing 1% glucose and increasing concentrations of MG (i. e., 40 μM, 80 μM, 400 μM). While all strains were found to grow only very weakly on medium supplemented with 400 μM MG, the addition of low doses of MG (40 μM) had no effect on the growth rate of the wild type and the overexpression mutants *PaGlo2*_OEx and *PaGlo1*/*PaGlo2*_OEx. Under these conditions, MG seems not to lead to high molecular damage and affects mycelial growth. In contrast, *PaGlo1*_OEx mutants are characterized by an increased growth rate compared to the wild type (+ 7.7%). Moreover, the Δ*PaGlo1* strain is unable to grow at all on PASM 1% glucose + 40 μM MG demonstrating an essential role of PaGLO1 to detoxify MG. The same tendency is observed when higher MG doses (80 μM) are applied, but on this medium *PaGlo1*_OEx strains do not show an elevated mean growth rate (Table 1).

**Table 1.** Analysis of growth rates. The mean growth rate of wild type isolates was set to 100%. Differences to the wild type are given in percentage of change. Individual differences of WT growth rates observed in experiments conducted at different times are due to slight environmental variations. At least three isolates were analyzed in each experiment. n. s.: not significant.

	PASM 1% glucose			PASM 2% glucose		
						
strain	mean growth rate [cm/d]	change [%] WT = 100%	p value	mean growth rate [cm/d]	change [%] WT = 100%	p value
WT	0.58			0.64		
*PaGlo1*_OEx	0.58	0	n. s.	0.63	− 1.6	n. s.
*PaGlo2*_OEx	0.63	+ 7.9	n. s.	0.65	+ 1.6	n. s.
						
WT	0.55			0.64		
*PaGlo1*/*PaGlo2*_OEx	0.53	− 3.6	n. s.	0.63	− 1.6	n. s.
						
WT	0.51			0.52		
Δ*PaGlo1*	0.55	+ 7.8	n. s.	0.57	+ 8.8	n. s.

	PASM 1% glucose + 40 μM MG			PASM 1% glucose + 80 μM MG		
						
strain	mean growth rate [cm/d]	change [%] WT = 100%	p value	mean growth rate [cm/d]	change [%] WT = 100%	p value
WT	0.52			0.52		
*PaGlo1*_OEx	0.56	+ 7.7	< 0.01	0.54	+ 3.8	n. s.
*PaGlo2*_OEx	0.54	+ 3.8	n. s.	0.48	− 7.7	n. s.
Δ*PaGlo1*	no growth	− 100.0	< 0.01	no growth	− 100.0	< 0.01
						
WT	0.58			0.53		
*PaGlo1*/*PaGlo2*_OEx	0.57	− 1.7	n. s.	0.56	+ 5.7	n. s.

**Table 2.** Analysis of median lifespans. The median lifespan of wild type isolates was set to 100%. Differences to the wild type are given in percentage of change. Individual differences of WT lifespans observed in experiments conducted at different times are due to slight environmental variations. The number of analyzed isolates is shown in parentheses next to the median lifespan. n. s.: not significant.

	**PASM 1% glucose**			**PASM 2% glucose**		
						
**strain**	**median lifespan [d]**	**change [%] WT = 100%**	**p value**	**median lifespan [d]**	**change [%] WT = 100%**	**p value**
WT	16.2 (n=21)			18.8 (n=16)		
*PaGlo1*_OEx	16.5 (n=62)	− 6.8	n. s.	16 (n=46)	− 14.9	< 0.01
*PaGlo2*_OEx	17.3 (n=35)	− 2.3	n. s.	17.7 (n=22)	− 5.8	n. s.
						
WT	15.2 (n=21)			15.1 (n=14)		
*PaGlo1*/*PaGlo2*_OEx	15.5 (n=21)	+ 0.2	n. s.	15.8 (n=16)	+ 4.6	< 0.05
						
WT	14 (n=20)			18 (n=20)		
Δ*PaGlo1*	14 (n=20)	0	n. s.	14 (n=20)	−22.2	< 0.001

Taken together, two of the transgenic strains generated in this study show a different growth rate than the wild type under certain conditions. Demonstrating the need for PaGLO1 in the efficient detoxification of MG, Δ*PaGlo1* fails to grow on MG-supplemented medium. The *PaGlo1*_OEx mutant is characterized by an increased mean growth rate when cultivated on PASM 1% glucose + 40 μM MG.

### Determination of median lifespan

To analyze whether or not the modification in the abundance of enzymes of the glyoxalase system affects aging, we compared the lifespan of the newly generated transgenic strains to those of the wild type. Meiotic offspring resulting from crosses between transformants and the wild type were isolated. The identity of strains was verified by growth selection on hygromycin B (overexpression strains) or phleomycin (Δ*PaGlo1* mutant) containing medium. Subsequently, survival curves were recorded by measuring the lifespan of the isolates in race tubes with synthetic medium containing either 1% glucose or 2% glucose (Table 2).

**Figure 3.**
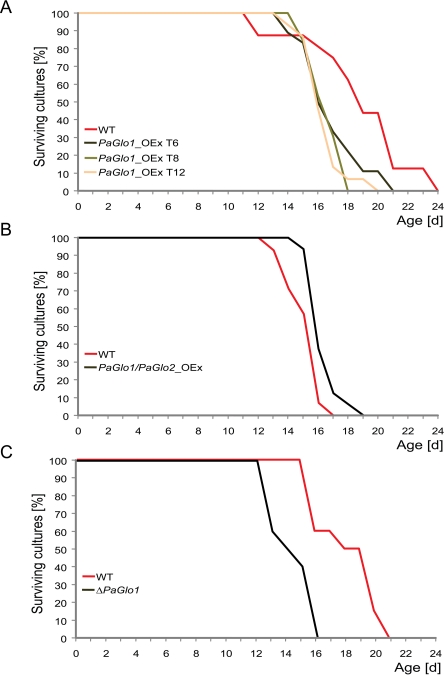
Survival curves of transgenic glyoxalase strains on PASM 2% glucose **A**Meiotic offspring of the three *PaGlo1*_OEx transformants T6, T8 and T12 is significantly short-lived compared to the WT (median lifespan: 16.2 d vs. 18.8 d). **B***PaGlo1*/*PaGlo2*_OEx double mutant shows a moderate but significant increase of the median lifespan (15.8 d vs. 15.1 d). **C***PaGlo1* deletion strains are negatively affected which is demonstrated by their shortened lifespan compared to the WT (14 d vs. 18 d). Individual differences of WT growth rates observed in experiments conducted at different times are due to slight environmental variations.

**Figure 4.**
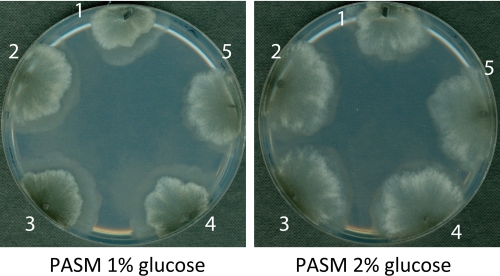
Phenotypic comparison between the wild type and transgenic glyoxalase strains Mycelia were grown for 4 d on PASM 1% glucose (left) and PASM 2% glucose (right). The morphology of the transgenic glyoxalase strains is identical to the wild type. 1: wild type; 2: *PaGlo1*_OEx; 3: *PaGlo2*_OEx; 4: *PaGlo1*/*PaGlo2*_OEx; 5: Δ*PaGlo1*.

On PASM containing 1% glucose no significant differences in median lifespan of the glyoxalase mutants were found compared to the wild type (Table 2). In marked contrast, on PASM containing 2% glucose *PaGlo1*_OEx, *PaGlo1*/*PaGlo2*_OEx and Δ*PaGlo1* strains are characterized by clear differences in lifespan (Figure 3, Table 2). In comparison to the wild type, *PaGlo1*_OEx and Δ*PaGlo1* strains are characterized by a significant reduction in lifespan of 14.7% and 22.2%, respectively (Figure 3 A, C). In contrast, *PaGlo1*/*PaGlo2*_OEx shows a moderate (+ 4.3%) but statistically significant increased median lifespan (Figure 3 B). Notably, the double mutant has a ‘healthy’ phenotype with no impairments in mycelial growth, pigmentation or the formation of aerial hyphae (Figure 4).

Collectively, the results of this series of experiments demonstrate that the lifespan of specific *P. anserina* glyoxalase mutants (i.e., *PaGlo1*_OEx, *PaGlo1*/*PaGlo2*_OEx and Δ*PaGlo1*) is influenced on medium containing elevated amounts of glucose.

## DISCUSSION

The central aim of this project was to study the effects of the glyoxalase system on *P. anserina* aging. Towards this end, we created and characterized transgenic strains in which the genes coding for the core components of the glyoxalase system, glyoxalase I and II, are either overexpressed or deleted. Since it is known from humans that hyperglycaemia caused by diabetes [25, 26] as well as increased glucose levels in the diet of nematodes [24] result in the formation of increased cellular MG levels we investigated the effect of the genetic modulation of the *P. anserina* glyoxalase systems on growth media supplemented with elevated levels of glucose (2% instead of 1%) and on media containing MG.

Median lifespan of Δ*PaGlo1* is strongly reduced on growth medium containing high levels of glucose (PASM with 2% glucose). Although not directly shown in our study, it is likely that the elevated levels of glucose lead to a concomitant increase of MG within the cell. Deletion of *PaGlo1* leads to hyper-sensitivity against MG. When added to the growth medium, MG efficiently kills Δ*PaGlo1* strains, demonstrating the importance of PaGLO1 to detoxify this compound.

*PaGlo1* overexpression strains are also short-lived compared to the wild type when grown on PASM containing 2% glucose. A possible reason for this behaviour is depletion of the reduced form of the tripeptide glutathione (GSH) which is directly involved in the detoxification of reactive oxygen species. With the role of GSH as one of the most important antioxidants within the cell and an essential cofactor of enzymes like glutathione peroxidase, glutathione-S-transferase and glutaredoxin it is not surprising that diminished levels of this compound are associated with a number of diseases, e. g. Alzheimer's disease [27] and Parkinson's disease [28]. MG needs to be conjugated with GSH to form a so-called hemithioacetal (HTA). HTA is then converted to S-lactoylglutathione by glyoxalase I. If high amounts of PaGLO1 are present, it is possible that a substantial fraction of available GSH is converted to S-lactoylglutathione which is not able to fulfill the role of a cellular antioxidant. It is important to note that our immunodetection analyses show that indeed very high levels of PaGLO1 are present within the *PaGlo1*_OEx isolates. This is not surprising because we used a strong constitutive promoter (0.56 kBp fragment of a metallothionein gene promoter). In contrast, in the study by Morcos et al. (2008), the PaGLO1 homolog CeGLY1 was overexpressed under the control of a weaker promoter in transgenic nematodes. The authors reported a beneficial effect on lifespan by overexpression of *CeGly1*. Moreover, they overexpressed a fusion gene containing the ORF for the *Gfp* gene rather than the native gene. Consequently, the data of that study cannot be directly compared with our results. In our study, sole overexpression of *PaGlo1* was not sufficient to improve lifespan. Only when *PaGlo2* is also overexpressed, a beneficial effect on median lifespan is observed. This strengthens our hypothesis that GSH depletion might be the crucial problem in *PaGlo1*_OEx strains, because increased PaGLO2 levels facilitate release of bound GSH from S-lactoylglutathione (*PaGlo2* catalyses the hydrolysis of S-lactoylglutathione to form lactate and GSH).

Similar findings demonstrating that forced expression of both genes encoding the components of the glyoxalase system is beneficial for survival of tobacco under salt stress were reported by Singla-Pareek et al. (2003) [29]. Moreover, *Gly1*/*Gly2* overexpressing tobacco mutants were also more resistant against exogenous MG. Interestingly, these mutants also produce less MG than their WT counterparts [30]. They contain higher levels of GSH and are capable to maintain higher GSH/GSSG ratios under stress situations. We hypothesize that a higher GSH/GSSG ratio in the *PaGlo1*/*PaGlo2*_OEx strains might represent an important contributing factor to an improved lifespan under high glucose conditions which favour increased production of MG. It is well known that the GSH/GSSG ratio in mitochondria is of key importancefor cellular survival [31]. Under non-stress conditions, mitochondrial GSH is able to cope with the oxidative stress generated in these organelles as a by-product of oxidative metabolism. However, depletion of GSH below a certain threshold ultimately leads to increased ROS-mediated damage to mtDNA, proteins and lipids like cardiolipin which is a component of the inner mitochondrial membrane. Oxidized cardiolipin is not able to bind one of the key factors of apoptosis, cytochrome-c [32]. Moreover, oxidized cardiolipin was demonstrated to maximize BAX-mediated pore formation, a further hallmark of apoptosis [32]. Apoptosis induction is also observed in the terminal stage of life in *P. anserina* [33]. Although BAX homologues are not identified in *P. anserina* yet, several other key factors involved in regulation of sensitivity towards apoptosis elicitation are found in this ascomycete (e. g., metacaspases [33], apoptosis-inducing factors and homologues [34], the mitochondrial fission protein PaDNM1 [7, 8] and the mitochondrial peptidyl prolylcis, transisomerase cyclophilin D [35, 36]). Therefore, it is tempting to speculate that overexpression of *PaGlo1* and *PaGlo2* leads to increased resistance to cellular damage and apoptosis induction, resulting in a prolonged lifespan without physiological impairments.

In conclusion, here we described the glyoxalase system in *P. anserina* as a novel lifespan determining factor in the complex molecular network involved in aging and lifespan control of this aging model. This experimentally well tractable model organism with a small, completely sequenced genome [21, 37] now offers excellent opportunities to further investigate the role of MG and the glyoxalase system as well as the glutathione metabolism on stress resistance and aging. Also, it appears to be promising to more carefully investigate and develop strategies to induce the glyoxalase system in genetically non-manipulated strains. Such interventions, e.g. via the application of mild stress conditions, may be of relevance also for influencing the aging process of higher organisms including humans.

## METHODS

### *P. anserina* strains and cultivation.

Overexpression strains *PaGlo1*_OEx, *PaGlo2*_OEx, *PaGlo1/ PaGlo2_*OEx and the *PaGlo1* deletion strain Δ*PaGlo1* are derived from wild type strain ‘s’ [38]. If not noted otherwise, the *P. anserina* isolates used in this study were grown on synthetic medium (PASM 1% glucose or PASM 2% glucose, respectively) in an incubation chamber at 27 °C. Composition of PASM (per litre): 0.5 g KH_2_PO_4_, 0.6 g K_2_HPO_4_, 1 g urea, 2.5 μg biotin, 50 mg thiamine, 5 mg citric acid x H_2_O, 5 mg ZnSO4 x 7 H_2_O, 1 mg Fe(NH_4_)_2_(SO_4_)_2_ x 6 H_2_O, 0.25 mg CuSO_4_ x 5 H_2_O, 0.05 mg MnSO_4_x H_2_O, 0.05 mg H_3_BO_3_, 0.05 mg Na_2_MoO_4_ x 2 H_2_O. For the determination of resistance againstmethylglyoxal (MG), MG concentrations of 40 μM and 80 μM were used in PASM 1% glucose. MG stock solution (40% in H_2_O) was purchased from Sigma Aldrich, St Louis, MO.

### *P. anserina* lifespan and growth rate analysis.

Ascospores from the wild type strain ‘s’ and the transgenic strains were isolated and germinated on cornmeal agar supplemented with 60 mM ammonium acetate. After three days of incubation, a small piece cut from the juvenile mycelium was placed into a race tube containing 50 ml PASM 1% glucose or PASM 2% glucose, respectively. Growth was measured until the isolate stopped to grow. Such cultures were defined as ‘dead’. The elapsed time to this point was recorded as the lifespan of the corresponding isolate. The median lifespan is defined as the time point when 50% of the analyzed cultures have died. Growth rates were determined by dividing the length of the mycelia by the time it took to grow. Individual differences in growth rate and lifespan of the WT controls observed in experiments conducted in independent experiments at different times are due to slight environmental variations. Therefore, in each individual experiment, freshly prepared wild type isolates were used as control.

### Homology analyses.

Homology between related proteins was determined using the BLOSUM62 algorithm implemented in ‘Clone Manager Suite 6’ (Scientific and Educational Software, Cary, NC).

### Constructs.

To overexpress *PaGlo1*in *P. anserina*wild type, the gene including the terminator region was amplified using primers XbaI-Glx1-f (5'- AATCTAGAATGGCGTCGACTACTGATG −3') and PstI-Glx1-r (5'- AACTGCAGTGTGACCCATCAGTT ATCC −3'). The PCR fragment was cloned after digestion into the *Xba*I/*Pst*I site of vector pExMthph [39] containing a 0.56 kbp promoter fragment of the metallothionein gene, *PaMt1* [40]. The resulting vector (termed pGlx1Ex) was transformed into wild type protoplasts. For overexpession of *PaGlo2* in the genetic background of the wild type, the gene including the terminator region was amplified using primers BamHI-Glx2-f (5'-AAGGATCC ATGCATATCCGGTCTATT CC-3') and HindIII-Glx2-r (5'-AAAAGCTT AGCGAA CCATAAAATCCG-3'). The PCR fragment was cloned after digestion into the *Bam*HI/*Hin*dIII site of vector pExMthph. The resulting plasmid (termed pGlx2Ex) was transformed into wild type protoplasts.

Deletion of *PaGlo1* in wild type strain ‘s’ was performed according to a previously published method [41]. First, small flanking regions of the *PaGlo1* gene were amplified using for the 5' flank oligonucleotides KoGlx1 (5'-TTGGTACC CAACTTGTCACTATCAGC −3') and KoGlx2 (5'-CCCAAGCTT AAATTCTCATT CATTAGC-3'), and KoGlx3 (5'-AAACTAGT GAAGGGCGGATATGCAGG-3') and KoGlx4 (5'- AAGCGGCCGCTACACATGGAAGGAGTGG-3') for amplification of the 3' flank. The 5' fragment was digested with *Kpn*I and *Hin*dIII and ligated into plasmid pKO3. The resulting plasmid was named pGlx1Ko1. The 3' fragment was digested with *Bcu*I and *Not*I and ligated into pGlx1Ko1. The resulting plasmid was termed pGlx1Ko2 and contains both fragments flanking a resistance cassette bearing phleomycin and blasticidin marker genes for fungal and bacterial selection, respectively. The resistance cassette with the flanking regions was excised by restriction with *Not*I and *Kpn*I and used to transform *E. coli* strain KS272 bearing the plasmid pKOBEG [42], which contains the ‘red’ region of bacteriophage lambda, and the cosmid 3A7 [43] bearing the *PaGlo1* locus. Homologous recombination between the flanks of the resistance cassette and cosmid 3A7 lead to generation of cosmid Δ*Glx*1#3A7, which contains the phleomycin-blasticidin cassette with large flanking genomic regions. The cosmid was isolated and used to transform *P. anserina* wild type strain ‘s’. Positive transformants (i.e., *PaGlo1* deletion strains) were selected by growth on phleomycin containing medium. The cosmid also bears a hygromycin B resistance cassette, which is integrated upon its ectopic integration into the genome. Therefore, positive transformants must not be able to grow on medium supplemented with hygromycin B.

### DNA sequencing.

DNA sequencing was performed by ‘Scientific Research and Development GmbH’, Oberursel, Germany.

### *P. anserina* transformation.

The constructs used in this study were transformed into *P. anserina* protoplasts according to previously published protocols [44, 45]. 5*10^7^ protoplasts were used in each transformation.

### Total protein extraction from *P. anserina.*

Total proteins were isolated by grinding approximately 3-4 g of wet mycelium in liquid nitrogen. The mycelial powder was dissolved in 1 mL extraction buffer (5 mM EDTA, 50 mM Na_2_HPO_4_, 1/100 Protease Inhibitor Cocktail [Calbiochem, Nottingham, UK], pH 7.5) and disrupted by vortexing cycles. The sample was centrifuged (25700 g, 10 min, Sorvall GSA SLA-7500 rotor) and the concentration of the supernatant was determined according to a modified protocol as described by Bradford using Roti-Nanoquant (Roth, Karlsruhe, Germany).

### Generation of antibodies.

Antibodies directed against components of the *P. anserina* glyoxalase system were newly generated: α-PaGLO1 raised against a PaGLO1 specific synthetic peptide ([Ac]-CVQNERFADKANF-[OH]; New England Peptide, Gardner, MA) corresponding to amino acids 302-313 and α-PaGLO2 raised against a PaGLO2 specific synthetic peptide ([Ac]-CFTIGDEKQHNV-[amide]; New England Peptide, Gardner, MA) corresponding to amino acids 213-223.

### SDS-PAGE and Western blot analysis.

100 μg of mitochondrial protein was incubated at 95 °C for 10 min in loading buffer (0.1 M Tris [pH 6.8], 6% SDS, 6% glycerol, 0.6 M ?-mercaptoethanol, 0.08% bromophenolblue) and was subsequently separated by using either 10 or 15% SDS-PAGE, respectively. After electrophoretic separation, proteins were transferred to a PVDF membrane (Immobilon-FL, Millipore, Schwalbach, Germany) using an electro-blotting system (Bio-Rad, Munich, Germany). Transfer membranes were incubated in blocking buffer (Li-Cor, Lincoln, NE, USA) for 1 h at RT and subsequently probed with the polyclonal *P. anserina* PaGLO1 antibodies (α-PaGLO1) (1:2000, overnight, 4 °C) or the polyclonal *P. anserina* PaGLO2 antibodies (α-PaGLO2) (1:2000, overnight, 4 °C). Labelling was detected with secondary antibodies (IRDye conjugated goat-α-rabbit [Li-Cor, Lincoln, NE, USA], 1:15000, 1 h, RT) and scanning the blots with an Odyssey infrared scanner (Li-Cor, Lincoln, NE, USA). As a loading control, transfer membranes were either treated with polyclonal *P. anserina* PaPRE3 antibodies directed against the β1-subunit of the 20S proteasome (1:2500, overnight, 4 °C) or stained with Coomassie Blue.

### Statistical analysis.

Statistical analysis of the results was performed by applying the Wilcoxon test (two-tailed).
